# Extracellular Heat Shock Proteins as Therapeutic Targets and Biomarkers in Fibrosing Interstitial Lung Diseases

**DOI:** 10.3390/ijms22179316

**Published:** 2021-08-27

**Authors:** Julie Tanguy, Lenny Pommerolle, Carmen Garrido, Martin Kolb, Philippe Bonniaud, Françoise Goirand, Pierre-Simon Bellaye

**Affiliations:** 1 Team HSP-Pathies, INSERM U1231, 21000 Dijon, France; julie_tanguy14380@hotmail.fr (J.T.); lenny.pommerolle@u-bourgogne.fr (L.P.); Carmen.Garrido-Fleury@u-bourgogne.fr (C.G.); philippe.bonniaud@chu-dijon.fr (P.B.); francoise.goirand@u-bourgogne.fr (F.G.); 2 Centre de Référence Constitutif des Maladies Pulmonaires Rares de l’Adultes de Dijon, OrphaLung Network (RespiFil), University Hospital of Burgundy, 21000 Dijon, France; 3 Firestone Institute for Respiratory Health, McMaster University, Hamilton, ON L8S 4L8, Canada; kolbm@mcmaster.ca; 4 University of Burgundy and Franche-Comté, 25000 Besançon, France; 5 Preclinical Imaging and Radiotherapy Platform, Nuclear Medicine Department, Centre George-François Leclerc, 21000 Dijon, France

**Keywords:** extracellular HSP, interstitial lung diseases, biomarker, lung fibrosis, IPF, heat shock proteins

## Abstract

Interstitial lung diseases (ILDs) include a large number of diseases and causes with variable outcomes often associated with progressive fibrosis. Although each of the individual fibrosing ILDs are rare, collectively, they affect a considerable number of patients, representing a significant burden of disease. Idiopathic pulmonary fibrosis (IPF) is the typical chronic fibrosing ILD associated with progressive decline in lung. Other fibrosing ILDs are often associated with connective tissues diseases, including rheumatoid arthritis-ILD (RA-ILD) and systemic sclerosis-associated ILD (SSc-ILD), or environmental/drug exposure. Given the vast number of progressive fibrosing ILDs and the disparities in clinical patterns and disease features, the course of these diseases is heterogeneous and cannot accurately be predicted for an individual patient. As a consequence, the discovery of novel biomarkers for these types of diseases is a major clinical challenge. Heat shock proteins (HSPs) are molecular chaperons that have been extensively described to be involved in fibrogenesis. Their extracellular forms (eHSPs) have been recently and successfully used as therapeutic targets or circulating biomarkers in cancer. The current review will describe the role of eHSPs in fibrosing ILDs, highlighting the importance of these particular stress proteins to develop new therapeutic strategies and discover potential biomarkers in these diseases.

## 1. Introduction

Interstitial lung diseases (ILDs) is a group of respiratory lung diseases affecting the lung interstitium which may lead to progressive lung fibrosis and ultimately, respiratory failure. Recent studies estimate that 13–40% of ILDs evolve to a fibrosing phenotype [1]. In others cases, pathology is spontaneously self-limited. Overall prevalence for progressive fibrosing ILDs is around 2.2–20 per 100,000 in Europe and 28 per 100,000 in the USA [1]. 

ILDs are typically assigned to many disease categories for classification and management purposes, roughly on the basis of a known underlying disease (e.g., pulmonary fibrosis associated with rheumatoid arthritis), an inciting agent (e.g., pneumoconiosis or drug side effect), or the absence of a known cause (idiopathic ILDs). Idiopathic ILDs include a large panel of diseases including idiopathic forms such as acute interstitial pneumonia (AIP), nonspecific interstitial pneumonia (NSIP) or idiopathic pulmonary fibrosis (IPF) [2], the most common form of fibrotic ILD [3]. On the other side, several diseases can induce pulmonary complications of known origin that may lead to progressive fibrosing ILDs in their most severe form [3]. For instance, a significant proportion of patients with systemic inflammatory/autoimmune disorders such as rheumatoid arthritis (RA), and systemic sclerosis (SSc) can develop ILDs and pulmonary fibrosis. Among all patients with fibrotic ILDs other than IPF, 13 to 40% have a progressive fibrosing phenotype [4]. Sarcoidosis (SA) is an idiopathic granulomatous disease that affects most commonly the lung and can evolve into fibrotic ILD [5]. The prognosis of idiopathic ILDs and, particularly, IPF, is poor and fibrotic ILD complications of underlying diseases such as RA and SSc are usually a marker of poorer prognosis in comparison to patients without lung involvement. As a consequence, a better understanding of the underlying mechanisms involved in lung fibrogenesis as well as the discovery of novel biomarkers for this type of disease is a major clinical challenge.

While fibrogenesis is different depending on the initiating insult and the affected organ, many of the major steps in its pathogenesis are similar [6]. Epithelial cell damage and apoptosis are thought to be major early events during fibrogenesis with subsequent recruitment and activation of inflammatory cells and an important secretion of pro-fibrotic cytokines such as PDGF, TGF-β and IL-10. PDGF is known to stimulate fibroblast proliferation and migration whereas IL-10 is an anti-inflammatory cytokine that promotes fibrosis by stimulating macrophage recruitment [7,8]. Such a pro-fibrotic microenvironment leads to the activation of neighboring cells and drives the progression of fibrosis. The hallmark of pulmonary fibrosis is an abnormal and massive increase in extracellular matrix (ECM) deposition, mainly collagen, which disrupts the alveolar architecture. Transforming Growth Factor-β1 (TGF-β1) is a key cytokine involved in the process of fibrogenesis as it plays a pivotal role during fibrosis in many different organ systems. TGF-β1 causes myofibroblast proliferation and differentiation, and increases the synthesis of ECM components such as collagen and fibronectin. Transient adenoviral vector-mediated gene transfer of active TGF-β1 (AdTGF-β1^223/225^) in rat or mouse lungs leads to progressive and severe fibrosis [9]. Moreover, it has been shown that this growth factor induces the transformation of alveolar epithelial cells into cells with a myofibroblastic phenotype, a process called Epithelial-to-Mesenchymal Transition (EMT) [10]. Furthermore, TGF-β1 levels are increased in fibrotic lung tissue from patients affected with pulmonary fibrosis [11]. 

**Figure 1:**
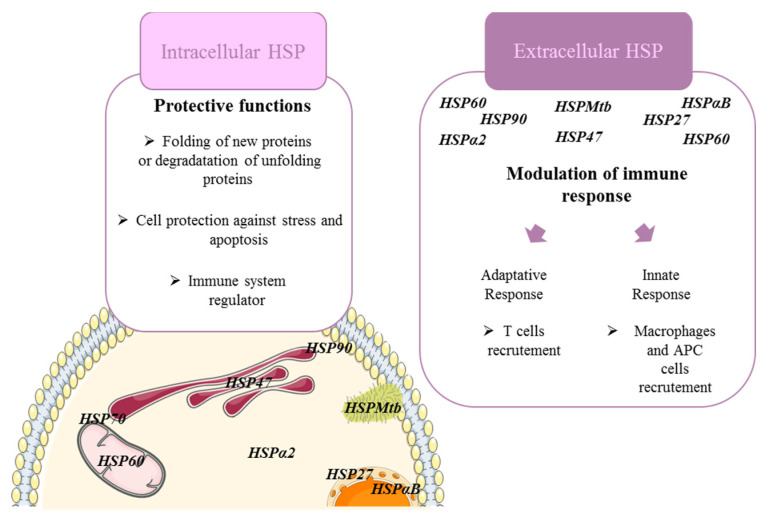
Schematic representation of HSPs’ biological functions. All HSPs mentioned are found in both intracellular and extracellular compartments.

Heat shock proteins (HSPs) are upregulated in cells following various stresses (hypothermia/hyperthermia, hypoxia, ionizing radiation, cytotoxic drug exposition (alkylating agents, antibiotics, antimetabolites, etc.)). They are classified in high molecular weight HSP (HSP110/HSPH, HSP90/HSPC, HSP70/HSPA, HSP60/Cpn and HSP47/DNAJ) and the small HSP (HSP27/HSPB1 and αβ-crystallin/HSPB5) [12] (Table 1). HSPs are molecular chaperones that are mainly involved in the folding of newly synthesized proteins to promote their stabilization, correct cellular location and proper turnover. During stress, the accumulation of misfolded proteins enhances the formation of aggregates, which disrupt the cellular machinery and can lead to cell death. The presence of HSPs ensures either the correct folding of misfolded proteins or, if refolding is impaired, they promote their degradation through the proteasome system [13] (Figure 1). In addition, HSPs have been extensively implicated in the pathogenesis of cancer. HSPs take part in several molecular mechanisms thereby favoring oncogenesis, tumor progression and/or cancer cell resistance. They inhibit key effectors of apoptosis machinery at the pre- and post-mitochondrial level, such as HSP27 associating cytochrome C, HSP90 inhibiting adaptor molecule apoptotic protease activation factor-1 (Apaf-1) or HSP70 associating Apaf-1 and apoptosis inducing factor (AIF). Thus, they have been implicated in cellular proliferation, immune modulation and tumor invasion, which is facilitated furthermore by their proangiogenic properties. Accordingly, HSPs appeared to be beneficial for cancerous cells and thereby deleterious for patients affected by a wide range of cancer types. Their depletion induces the regression of the tumors [14]. Therefore, the inhibition of HSPs appears to be of great interest to improve the efficiency of chemotherapy and the disease outcome, and a large number of inhibitors targeting HSP are currently developed or clinically tested for cancer treatment [15].

The intracellular role of HSPs in fibrogenesis has largely been investigated [16]. HSP47 is the most studied HSP in fibrogenesis due to its specific role on collagen synthesis and secretion [17,18,19]. Hagiwara et al. demonstrated that HSP47 inhibition by antisense-oligonucleotide reduced lung injury and collagen deposition as well as improved lung morphology and function in several experimental models [20,21,22]. In addition, HSP90 has been shown to favor TGF-β1 signaling pathway by stabilizing TGF-βRII, thus promoting myofibroblast activation and ECM production [23,24]. Some HSP90 inhibition strategies have been shown efficient to reduce lung fibrosis in mice models [23]. On the contrary, HSP70 has been shown to protect from lung fibrosis. Knock-out mice for HSP70 presented higher sensitivity to bleomycin-induced lung fibrosis [25] and HSP70 induction via geranylgeranylacetone has been shown to prevent lung fibrosis in animal models [26]. Small HSPs have also been involved in the process of fibrogenesis. HSP27 was described as a chaperon protein to Snail, an EMT transcription factor, able to enhance the TGF-β1 signaling pathway [27]. Its inhibition with OGX-427, an HSP27-targeted oligonucleotide antisense, was shown to inhibit fibrosis in animal models. In addition, αβ-crystallin was described to be essential for the activation of TGF-β1 signaling through the enhancement of the nuclear translocation of Smad4 [28] and the sequestration of TRIM33, a negative regulator of lung fibrosis [29]. 

Although HSPs were mainly studied for their intracellular cytoprotective and chaperon role, recent work highlighted a major role of secreted HSPs which interact with immune cells and act as immunomodulators (Figure 1). 

Furthermore, growing body of evidence has shown that extracellular HSP (eHSP) could be used as circulating biomarkers, mainly in cancer [30]. Extracellular HSPs were described to be involved in tumor progression and metastasis [31]. For example, HSP70 serum level has been shown to correlate with tumor volume in non-small cell lung cancer [32]. HSP27 has also been extensively studied in various cancers including breast, ovarian, or colorectal cancer in which an increase in its extracellular level has been associated with poor prognostics [33,34,35]. In the tumor microenvironment, extracellular HSP27 (eHSP27) was able to activate TLR3 receptors and thereby to promote angiogenesis [36]. Recent studies showed that eHSPs could also be found in extracellular vesicles (EVs) and could represent new perspectives for diagnosis and therapeutic strategies [30]. For example, a recent study showed that the concentration of circulating HSP70-positive exosomes in lung and breast cancer patients was significantly higher compared to healthy volunteers and were useful to discriminate metastatic from non-metastatic patients [37].

**Figure 2:**
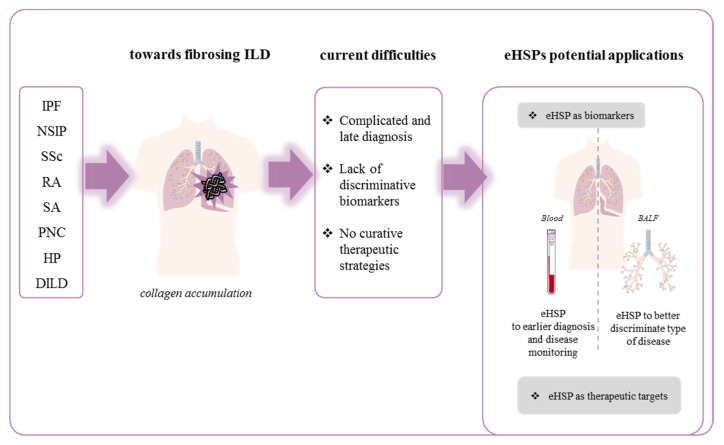
eHSPs are a promising biomarker and therapeutic target in ILDs. IPF: idiopathic pulmonary fibrosis; NSIP: nonspecific interstitial pneumonia; SSc: systemic sclerosis; RA: rheumatoid arthritis; SA: sarcoidosis; PNC: pneumoconiosis; HP: hypersensitivity pneumonitis; DILD: drug-induced lung disease.

The current review will describe the role of extracellular HSPs in several lung disorders involving ILDs and progressive lung fibrosis highlighting the importance of this particular stress proteins to develop new therapeutic strategies and discover potential biomarkers in fibrosing ILDs (Figure 2 and Table 2).

## 2. Heat Shock Proteins Modulate Immune Cell Functions in Fibrosing ILDs

Even if the origin of inflammation leading to fibrosis in ILDs depends on the pathogenesis of each diseases, similar sub-types of immune cells and cytokines/chemokines are involved in all cases. Briefly, it is clear that cells of both the innate and adaptive immune system such as macrophages, APC cells, CD4+ T-cells (Th1, Th2, Th17) are linked to the pathogenesis of fibrosing ILDs [63,64] (Figure 1). Oversimplified M1/M2 macrophage polarization nomenclature can help to understand how macrophages contribute to fibrosis. M1-like macrophages have been found to promote fibrosis by secreting pro-inflammatory IL-6, IL-12 and TNF-α as well as promoting the Th17 and neutrophilic immune response [65,66,67,68]. In autoimmune or inflammatory fibrosing ILDs, macrophages promote secretion of pro-inflammatory cytokines, ROS, stimulates Th1/Th17 response and inhibits regulatory cells (e.g., RA, SA, hypersensitivity pneumonitis, pneumoconiosis) [69]. M2-like macrophages have been linked to fibrotic processes through the promotion of CC ligand (CCL) 2 and CC ligand 17 and particularly by increased secretion of TGF-β, IL-10, and IL-33; three well-known pro-fibrotic cytokines. These suppressive cells, implicated in wound healing, stimulates Treg/Th2 expansion and restrains T-cell activation. In SSc-ILDs, macrophages seem to be programmed to differentiate in M2 phenotype through TLR2-TLR4 signaling and promote fibrosis [70]. In addition, in response to interactions with pathogen-associated molecular patterns or danger-associated molecular patterns and TLR2-TLR4 signaling, antigen-presenting cells (DC, neutrophils or macrophages) can also adopt fibrosing properties as seen in SA with HSPs from pathogens such as *Mycobacterium tuberculosis*. In IPF and SSc-ILD, increased production of CCL18 by APC cells promotes collagen production by lung fibroblasts [7]. On the contrary, the role of lymphocytes in fibrosing ILDs remains poorly understood. Nevertheless, an excessive accumulation of T lymphocytes has been observed in several pulmonary fibrotic diseases (SSC-ILDs and IPF) [71]. Moreover, a disturbance in Th1/Th2 balance with abnormal profibrotic Th2-polarized T-cell response has been associated with tissue damage and fibrosis in SSc-ILD and in IPF [72,73]. 

Immunomodulation by regulatory immune cells is crucial in dampening pathogenic immune responses and inhibiting the transition from inflammation to fibrosis. Therefore, many current treatments against fibrosis in all ILDs are based on immunoregulators such as glucorticoids and TNF inhibitors. It has been shown that extracellular or receptor-bound HSPs mediate immunological functions and immunomodulatory activity [74,75]. Therefore, modulating HSPs in the context of ILDs could help to restore a proper inflammatory environment and promote patients’ recovery by slowing down fibrosis. 

For instance, Hsp70, known to be involved in SSC-ILD, SA, RA, hypersensitivity pneumonitis or IPF promotes the production of anti-inflammatory cytokines and interacts with APC and macrophages by binding to their endocytic receptors resulting in the release of anti-inflammatory cytokine IL-10 that promotes fibrosis [76]. Moreover, eHSP70 has also been observed not only to induce the production of other pro-fibrotic cytokines such as IL-6 and TGF-β in auto-immune diseases but also to increase Th17 response in these diseases [77]. TLR2/4 receptors’ activation is the main characterized pathway through which eHSP70 may induce its inflammatory effects. Therapeutics strategies modulating HSP70 expression in tumor environment have been able to modulate regulatory phenotype of macrophages and promotes tumor regression [78]. One can imagine that in a same way, targeting eHSP70 expression in the lung could help to slow down fibrosis by modulating macrophage polarization and lung inflammation, damage and fibrosis. Similarly, Hsp90 involved in the pathogenesis of SSc/RA-ILDS or pneumoconiosis is known to promote wound healing and activate innate immune system through NFKB signaling [79,80]. Recently, Hsp90 inhibitors have been shown to ameliorate autoimmune RA, and lung inflammation by modulating macrophages, T and DC activity, and may contribute to develop new therapies against fibrosing ILDs [81]. Finally, HSPs from *mycobacterium tuberculosis* that participates to fibrosing SA, and environmental ILDs, are capable of eliciting important inflammatory immune response through TLR signaling to the extent that they have been labeled as “superantigens” [82]. Their properties can explain how they promote fibrosis through inflammation-induced damage to the lung. 

The complete mechanisms by which eHSPs modulate immunity and ILDs’ pathogenesis are yet to be understood. However, it is clear that they are a promising therapeutic target that can not only be used as a biomarker but also to modulate immune response in fibrosing ILDs (Figure 1).

## 3. Extracellular HSP and Fibrosing Diffuse Interstitial Lung Disease 

### 3.1. Fibrosing Idiopathic Diffuse Interstitial Lung Disease 

#### 3.1.1. Idiopathic Pulmonary Fibrosis

Idiopathic pulmonary fibrosis (IPF) is the most common and canonical chronic fibrosing ILD. It is a devastating disease with an unknown etiology and a median overall survival of approximately 5 years. This rare disease reaches a prevalence around 0.33 and 4.51 cases per 10,000 [83]. This pathology also affects more men than women and rather the elderly population (rarely under the age of 60) [3,84]. Aging is the main risk factor but genetic predisposition and environmental factors such as cigarette smoke, wood dust and air pollution may be involved [85]. To date, only two drugs, pirfenidone and nintedanib, have been shown to slow down disease progression and no other drug demonstrated efficiency to stop or reverse it [84,86]. 

HSPs and eHSPs were highlighted to take part in the pathogenesis of IPF and have been widely investigated [16]. Dong et al. demonstrated the usefulness of 1G6-D7, a monoclonal antibody specifically targeting eHSP90α, to attenuate bleomycin-induced lung fibrosis by interfering with LRP1-Erk signaling [87]. In 2018, Bellaye et al. confirmed the importance of eHSP90 as a potential circulating biomarker. They demonstrated a higher level of HSP90α in the sera of IPF compared to non-IPF patients in correlation with the worsening of fibrosis assessed by respiratory parameters (TLC, FVC and Fev1). In this study, HSP90α was also found increased in BALF (Broncho Alveolar Lavage Fluids) and sera from rats with AdTGF-induced lung fibrosis in correlation with collagen level in the lungs. This work also demonstrated a direct interaction between HSP90β (intracellular isoform) with LRP1 which seems to be essential for the stabilization of LRP1 in plasma membrane as well as to allow eHSP90α/LRP1 signaling and to promote myofibroblast differentiation and persistence [88].

Wallace et al. were the first to describe circulating autoreactive IgG against a 70–90 kDa alveolar epithelial cells’ antigen in IPF [38]. Recently, a higher concentration of anti-HSP70 IgG in sera of IPF patients compared to healthy volunteers was described. In this study, the presence of anti-HSP70 IgG was correlated with a poor prognosis in IPF patients and with the development of acute exacerbations [39], defined as increasing dyspnea and or hypoxemia within the preceding 30 days, and new radiographic infiltrates without other attributable cause of lung dysfunction [89]. They demonstrated that anti-HSP70 IgG isolated from IPF patients were able to induce lymphocyte and monocyte activation with an increase CXCL8 secretion associated with disease progression. In contrast, elevated HSP70 level in the serum of IPF patients compared to healthy subjects was observed by Mills et al. but without difference with other ILD patients. In this study, higher BALF levels of anti-HSP70 antibodies were also found in non-progressor IPF patients compared with progressors but no difference was observed in the antibody levels in the serum between these two groups of patients. These results support a rather beneficial role of intra-pulmonary anti-HSP70 antibodies which may promote a homeostatic immune response [40]. 

Regarding HSP47, the collagen-specific HSP, the level of protein and autoantibodies in the sera were not significantly elevated in IPF patients compared with RA or mixed connective tissue disease (MCTD) patients [41]. These results were further investigated by Kakugawa et al. who demonstrated that the serum level of anti-HSP47 was higher in NSIP than IPF patients. In this study, IPF patients did no longer present higher level of anti-HSP47 compared with healthy controls [42]. A study involving a small cohort showed an increase in HSP47 level in the serum of IPF patients with acute exacerbations, compared with stable IPF patients [43]. These results, which should be confirmed in a larger cohort of patients, suggest different mechanisms of regulation of circulating HSP47 between stable patients and patients with acute exacerbation. Up to date, findings are too scarce to support the usefulness of measurement of HSP47 or autoantibody against HSP47 circulating level in the monitoring of IPF patients (Figure 2 and Table 2). 

#### 3.1.2. Nonspecific Interstitial Pneumonia (NSIP)

Nonspecific interstitial pneumonia (NSIP) is often of unknown etiology but its development can also occur secondarily to a connective pathology or following toxin exposition [90]. Still poorly understood, the diagnosis of this disease requires the exclusion from all the other lung pathologies. Sometimes, the difference between nonspecific interstitial pneumonia and usual interstitial pneumonia (UIP), an imaging and histopathological pattern that characterized IPF, is even difficult to determine. Indeed, a recent work mentioned that an expansion of fibrotic alveolar walls and fibroblast foci could also be observed in NSIP without being immediately associated with a more deadly UIP [91]. NSIP has a better prognosis than IPF [92]. NSIP may present as an “inflammatory type” associated with an important lymphocytic tissue inflammation and good response to immunomodulating therapies including corticosteroids or a “highly fibrotic type” associated with lung fibrosis, no response to therapy, and rapid evolution towards progressive fibrosis [93]. As previously described in many fibrotic diseases, the expression of HSP47 is increased in type II pneumocytes and fibroblasts in NSIP in association with a poorer prognosis [55,56]. Additional work performed in sera of patients with different fibrosing lung diseases showed that higher levels of anti-HSP47 autoantibodies were found in idiopathic fibrosing NSIP patients than in patients with IPF or in healthy volunteers [42]. The therapeutic or prognostic value of this marker remains to be elucidated. No other eHSP has been investigated in NSIP and, due to their involvement in other fibrotic diseases, more studies are needed to provide new information on specific markers and therapeutic strategies in this disease (Figure 2, Table 2).

### 3.2. Fibrosing Diffuse Interstitial Lung Disease Associated with Connective Tissue (CTD-FLD)

#### 3.2.1. Systemic Sclerosis-Associated Interstitial Lung Disease (SSc-ILD)

Systemic sclerosis (SSc) is a rare chronic autoimmune disease of unknown cause characterized by diffuse fibrosis and vascular abnormalities in the skin, joints, and internal organs. Interstitial lung disease (ILD) affects approximately 30 to 50% of SSc patients and is in its progressive fibrosing form, the leading cause of death in SSc. SSc-ILD belongs to the connective tissue diseases (CTDs) and affects mostly young and middle-aged women [94,95]. This disease is characterized by severe tissue damage, which impairs epithelial cells that release several pro-fibrotic cytokines thereby promoting the differentiation of myofibroblasts. Moreover, these myofibroblasts together with remaining resident fibroblasts generate a global immune dysregulation by aggravating inflammation, promoting immune cells’ recruitment and fibrosis [96]. Some studies have shown that mesenchymal cells from SSc-ILD patients strongly upregulate the expression of collagen and other profibrotic genes’ expression such as ACTA2 or WIF1 [97]. However, most studies report that dysregulation of the immune system causes an accumulation of autoantibodies [98] responsible for the onset of SSc-ILD. Due to its importance in this pathology, the immune system is a major target to develop biomarkers and therapeutic tools. Current strategies targeting the immune system have been shown to slow the progression of the disease. Indeed, immunosuppressant combination therapies such as mycophenolate mofetil and cyclophosphamide are currently used to delay disease progression [98]. Moreover, recent clinical studies have shown that antifibrotic agents approved in IPF such as nintedanib may be indicated in progressive SSc-ILD to slow down fibrosis progression [99]. In most severe cases, both hematopoietic stem cell transplantation and lung transplantation are also discussed [95]. Due to the lack of available effective treatments, biomarker discovery is the next challenge in therapeutic care of SSc-ILD patients [100]. In recent years, several potential biomarkers and therapeutic targets have been proposed. Among them, some alveolar epithelial cell damage markers such as SP-D (surfactant protein D) and KL-6 (Krebs von den Lungen-6) have been described and are directly correlated with lung disease severity [101]. In this context, some HSPs have also been defined as circulating biomarkers. Luzina et al. showed in a mouse model of bleomycin-induced SSc-ILD that extracellular HSP70 (eHSP70) levels were enhanced in association with IL33, a maker correlated with pulmonary fibrosis severity in sera of SSc patients [44]. These studies also unveiled an increase in eHSP70 levels in BALF from patients with SSc-ILD and reinforce the interest in this extracellular HSP as a therapeutic and diagnostic tool [44]. Recently, Ogawa et al. revealed that eHSP70 level in patients’ sera correlated with pulmonary fibrosis and the modified Rodnan total skin thickness score (which scores the SSc stage in patients) [45]. 

Other studies have shown that eHSP90 was associated with increased inflammatory activity, worse lung functions, and severe skin involvement in patients with SSc. In this study, baseline HSP90 level in plasma was predictive of the 12-month change in DLCO (diffusing capacity for carbon monoxide) in SSc-ILD patients [46]. In addition, the expression of HSP27 also appears to be increased in the blood of SSc patients [47]. On the contrary, proteomic investigation performed in BALF from SSc patients with and without pulmonary fibrosis described a decrease in α-2 heat shock protein, specifically in patients with lung fibrosis, suggesting that individual HSPs may be differently regulated depending on specific diseases [48]. Taken together, these findings suggest that eHSPs are promising easily accessible and detectable biomarkers for SSc-ILD patients’ follow-up and could improve patient care. Nonetheless, little is known about the involvement of increased level of HSPs in SSc-ILD pathogenesis and further investigations are required to understand whether they can be potential therapeutic targets in SSc-associated lung fibrosis (Figure 2, Table 2).

#### 3.2.2. Rheumatoid Arthritis-Associated Interstitial Lung Disease (RA-ILD)

Rheumatoid arthritis (RA) is one of the main connective tissue diseases associated to ILD. Progressive lung fibrosis is a major complication of RA, which occurs in 30% of patients affected with this inflammatory joint pathology [102]. RA-ILD can further be divided according to its severity and prognosis into nonspecific interstitial pneumonia (NSIP) or usual interstitial pneumonia (UIP) which presents 1.6-fold higher risk of death. Moreover, RA is a gateway to opportunistic infections and drug toxicity which increases significantly disease-associated morbidity and mortality [103]. This pathology is characterized by a global inflammatory response in patients that progressively affects many tissues including the lungs. This disease is distinguished by the presence of autoantibodies not only in blood but also in the lungs. They are representative of a systemic inflammation characterized by the presence of inflammatory cytokines and chemokines. Increased level of TNF-α, IFN-γ, IL-6, IL-12, ROS, and other chemokines are observed with an enhanced recruitment of inflammatory macrophages and neutrophils that are also described in the pathogenesis of lung fibrosis [104]. Beyond glucocorticoids, disease-modifying antirheumatic drugs (DMARDs) like methotrexate, TNF-α inhibitors, and other emerging biotherapy are indicated and often efficient to treat the rheumatologic disease. Antifibrotics (nintedanib or pirfenidone) have been recently recommended in progressive lung fibrosis associated with RA [105]. However, level of evidence of their use is low [106]. Thanks to high-resolution computed tomography (HRCT) different researchers have been able to correlate ILD progression and mortality in RA-affected patients [105]. Unfortunately, HCRT is not an early diagnosis tool and can only be used when pulmonary symptoms are seen in patients with RA. Thus, tools that allow an early detection of patients at risk to develop ILD are needed and under investigation. Recently, several biomarkers have been proposed to evaluate ILD in RA patients. Among these markers, the gain-of-function MUC5B promoter variant involved in mucin production [107], KL-6, and other glycoproteins produced by pneumocytes are described as potential ILD biomarkers [108]. In this context of discovering predictive markers in RA-ILD, the search of autoantigens containing the amino acid citrulline in inflamed fluid seems to be a promising axis [109]. Previous work have identified the presence of anti-citrullinated HSP90 antibodies in patients’ sera and determined that they can help to distinguish RA-ILD from RA or IPF [49]. Furthermore, Harlow et al. have also identified the presence of citrullinated HSP90β that induced an autoreactive T-cell response in BALF from RA patients [110]. It has also been described that incubation of blood from RA-ILD patients with citrullinated HSP90β, induces IFN-γ (Th1 like cytokine) expression compared to blood from RA patients without ILD [111]. These studies suggested that citrullinated HSP90β could promote inflammation and may contribute to fibrosis in a RA-ILD context. This assumption needs to be further investigated and could promote citrullinated HSP90β as not only a predictive biomarker but also an interesting therapeutic target to treat RA-ILD. In classical RA, many other eHSPs have been also studied for their therapeutic potential [50]. For example, the dual (immunosuppressive or pro-inflammatory) role of eHSP70 has been notably observed in human peripheral blood mononuclear cells. Tukaj et al. demonstrated that HSP70 mediated IL-6 secretion and, consequently, an increase of Th17 and Th17/Treg ratio and a decrease in Th1 and Th1/Th2 ratio [112]. As these effects are associated with the promotion of fibrosis in lung tissue, studying eHSP70 implication in RA-ILD could provide new therapeutic strategies. Furthermore, in this constant quest for predictive biomarkers, a deeper investigation of the relevance of anti-HSP autoantibodies in RA-ILD may be interesting in order to anticipate and mitigate their pulmonary manifestations (Figure 2, Table 2). 

### 3.3. Sarcoidosis-Associated Interstitial Lung Disease (SA-ILD)

Lung are involved in 90 to 95% of cases of sarcoidosis, a pathology of unknown etiology, and patients present respiratory and systemic symptoms, including cough, chest pain, fatigue, musculoskeletal symptoms, fevers, and weight loss. Sarcoidosis-associated interstitial lung disease mortality is increasing over the years [113]. Even though little is known about the pathogenesis of this disease, some research showed that an unknown antigen presented by antigen-presenting cells (APCs) (such as macrophages, dendritic cells or AEC II) to naive T-cells could be responsible for its initiation. The polarization of the T lymphocytes into a Th1 phenotype might induce the recruitment of inflammatory macrophages and lymphocytes that proliferate and differentiate into a sarcoid granuloma able to initiate fibrosis. This peripheral fibrosis causes a massive deposition of collagen which obliterates the parenchymal tissue and constitutes the final stage of sarcoidosis [114]. Systemic corticosteroids are often the first line of treatment for SA but some other therapies such as methotrexate or TNF-alpha inhibitors can also be recommended [115]. Therapies used to treat SA generate multiple side effects, which is why discovery of new therapeutic agents are needed. In this context, it has been reported that some IgG against HSP70 but not HSP90 were increased in the sera of patients with pulmonary sarcoidosis [51]. Due to the similarities between sarcoidosis and tuberculosis, some studies suggested that *Mycobacterium tuberculosis* could be a promoting factor of SA-ILD [116]. Some bacterial HSP such as *Mycobacterium tuberculosis* (Mtb) HSP65, HSP70, and HSP16 were investigated in the context of SA. Mycobacteria antigens are recognized by the host immune system as PAMPs/DAMPs (pathogen-associated molecular patterns/damage-associated molecular patterns) and presented by antigen-presenting cells (APC). As a consequence, HSP*_Mtb_* are expressed on the surface of APC to be presented to T-cells and may promote an autoimmune response involved in SA. Dubaniewicz et al. demonstrated that HSP*_Mtb_* were present in lymph nodes from SA-ILD patients and participated at several stages of the disease’s development. HSP16*_Mtb_* was expressed at early stage whereas HSP70*_Mtb_* was expressed later and HSP65*_Mtb_* was continually expressed in capillary vessels in the lymph nodes [117]. Highly expressed HSP16*_Mtb_* would be the main actor to initiate the autoimmune response in SA. Moreover, it appears that HSP16*_Mtb_* maintained *M. tuberculosis* in a genetic dormant stage in patients and is believed to further promote the development of SA [52]. Further, HSP16*_Mtb_* inhibited nitric oxide synthase’s expression, antimicrobial activity, and induced monocyte apoptosis resistance, a feature of sarcoidosis, suggesting that HSP16*_Mtb_* may have a prominent role in sarcoidosis pathogenesis. Treatment of peripheral blood mononuclear cells from SA patients with Mtb-HSPs induced a strong upregulation of pro-inflammatory cytokines suggesting that these antigens could elicit the host’s immune response [54]. Mtb-HSPs signaled through specific receptors, mainly TLR2 and TLR4, and induced an inflammatory cascade that damaged tissues and contributed to the release of more pro-inflammatory mediators resulting in chronic inflammation and autoimmunity. Nevertheless, the inflammatory response induced by Mtb-HSPs is dependent on the genetic predisposition of the host [116].

In another study on patients diagnosed with sarcoidosis, it was shown that high levels of HSP65 and HSP70 were found in alveolar macrophages retrieved in BALF [53]. 

Besides, it is worthwhile to note that several inflammatory bowel diseases, also associated with granuloma, generate pulmonary complications [118]. For example, Crohn’s disease was described to mimic sarcoidosis with some patients presenting lymphocytic alveolitis in BALF with an elevated CD4/CD8 ratio, a highly specific marker of sarcoidosis [119]. As eHSP such as HSP90α or HSP60 are already described to be involved in inflammatory bowel diseases [120], it should be interesting to further explore their role as new biomarkers and therapeutic targets in the development of fibrosing lung complications in these types of diseases (Figure 2, Table 2).

### 3.4. Fibrosing Environmental Interstitial Lung Disease 

#### 3.4.1. Pneumoconiosis (PNC)

Pneumoconiosis are a group of disease caused by the inhalation of inorganic dust mainly observed in a professional environment. These ILDs are often associated with progressive fibrotic damage to the lung. Silicosis and asbestosis are the most common forms of pneumoconiosis [121]. Alveolar macrophages present inside the lung will respond to the inhalation of particles by inducing phagocytosis and initiating the inflammatory process, ultimately leading to collagen production and development of a fibrotic pneumoconiosis [122]. Currently, there is no effective treatment for this pathology. In this context, finding early disease markers may help to limit the evolution of pneumoconiosis into lung fibrosis. Yang et al. showed in coke oven workers that HSP70 level in plasma was increased proportionally to the dose of burned coal they were exposed to [57]. Surprisingly, in lymphocytes from coke workers, HSP70 expression was negatively proportional to patients’ exposure to particles. This may suggest that a high level of particles reduces HSP70 which may have a beneficial role on the disease progression, as previously shown in other fibrotic diseases. In peripheral blood lymphocytes from coke oven workers, several studies described the existence of three polymorphic forms of the HSP70 gene: HSP70–1 G190C, HSP70–2 G1267A, and HSP70-hom T2437C. People with homozygous HSP70–1 C/C genotype were the most susceptible to develop DNA damage inside their peripheral blood lymphocytes. Expression of HSP70 and evaluation of HSP70 polymorphism in circulating lymphocyte collected from the blood may represent a novel type of “liquid biopsy” instead of direct measurement of circulating HSPs. Thus, further investigation is needed to determine if HSP70 in plasma and/or in circulating lymphocytes can be used as a biomarker to protect employees from occupational pneumoconiosis and its complications [58]. Similarly, Zhang et al. determined that HSP90 was increased in blood lymphocytes from coke oven workers and this was dependent on the exposition to polycyclic aromatic hydrocarbons (PAHs [59]). These data suggest that HSP90 may also be involved in pneumoconiosis and may serve as a biomarker, as HSP70, in circulating lymphocytes. Interestingly, this study also highlighted that HSP60 and HSP27 expression in circulating lymphocytes were similar in coke oven workers and controls suggesting a specific interest of HSP70 and HSP90 in this disease [59]. Some others studies showed that the TGF-β1-dependent SMAD pathway and EMT could be promoted by exposition to silica. As EMT is one of the major processes in pneumoconiosis/fibrosis and HSPs are important regulators of EMT, one can hypothesize that modulating HSPs might limit fibrosis progression in pneumoconiosis [123]. Unfortunately, to date, no studies further investigated the role of eHSPs in silica-induced EMT. Lastly, several works have identified a relationship between coal workers’ pneumoconiosis and *Mycobacterium malmoense*, a nontuberculous mycobacteria [124]. Knowing that HSPs from *Mycobacterium* have been incriminated in ILD associated with sacroidosis, there is a potential interest, not yet investigated, to evaluate their expression and potential use as a biomarker in pneumoconiosis (Figure 2, Table 2).

#### 3.4.2. Hypersensitivity Pneumonitis (HP)

Hypersensitivity pneumonitis is an immune-mediated inflammatory lung disease caused by the inhalation of a large variety of antigens from animals, bacteria, fungi, plants, metals, and chemicals found in the environment [125]. These antigens are responsible for several specific well-described entities with, for instance, Farmer’s Lung Disease Bird Fancier’s Lung or metalworking-fluid hypersensitivity pneumonitis or hot tub lung. HP are classified into nonfibrotic and fibrotic phenotypes [125]. Early detection of the factors responsible for these diseases would allow to prevent their progression from acute forms to chronic fibrotic forms with poor prognosis [126]. Patients with HP present an increase in IL-17 potentially caused by a defective regulatory T-cell function that leads to an exacerbated immune response. In fibrotic HP patients, an important increase in T-helper 17 lymphocytes (CD4+) has been observed. This increase in pro-inflammatory Th17 cells, known for their implication in autoimmune disease or fibrotic mechanisms, could give additional explanation on the development of fibrosis during HP [127]. Concerning HP’s treatment or prevention, the most obvious recommendation is to limit exposure to previously described pathogens in a professional environment, but corticoids can be recommended to circumvent inflammation in patients with mild HP. In severe cases of HP, a lung transplantation can also be considered [128]. In order to improve the management and prevent serious forms of the pathology, several studies are investigating serological or BALF markers in context of HP. For example, KL-6, previously described in connective disease, was found to be increased during the acute exacerbation of HP and correlated with SP-D expression by different epithelial lung cells in this disease [129]. In this context, HSPs have been found to act as promising circulating markers. Racine et al. showed that alveolar macrophages (AMs) from challenged mice with antigens from *Saccharopolyspora rectivirgula* (SR) exhibited increased basal level of HSP70 compared to control mice. However, a second stimulation with SR-antigen on these AMs, did not further enhance HSP70 levels in sensitized mice compared to control mice, which showed much higher HSP70 levels [60]. The absence of HSP70 response to an additional stress in HP mice could be explained by the altered immunoregulatory activity observed during HP. Furthermore, AMs isolated in BALF from HP patients expressed higher levels of HSP70 than healthy patients. Altogether, these findings suggest that targeting HSP70 in circulating macrophages could modulate the dysregulated immune response. Interestingly, it has been reported that expression of α-2 heat shock glycoprotein is decreased in BALF from patient with fibrotic HP compared to HP patients with usual interstitial pneumonia phenotype [61]. Therefore, α-2 heat shock glycoprotein and HSP70 in circulating immune cells may be potential therapeutic targets and biomarkers that could be used to anticipate the evolution of this pathology (Figure 2, Table 2)

#### 3.4.3. Drug-Induced ILD

Drug-induced lung disease (DILD) is another set of ILD associated with an exposure. Kakugawa et al. [62] have evaluated serum HSP47 levels in patients with DILD. This study demonstrated that serum HSP47 levels were elevated in patients with DILD who had the worst outcomes among the different subgroups of DILD. Serum HSP47 levels also significantly correlated with various respiratory parameters. Interestingly, they show that serum levels of HSP47 in the group of patients requiring glucocorticoids were significantly higher than those in patients who experienced clinical improvement without glucocorticoid administration (Figure 2, Table 2). 

## 4. Conclusions

This review highlights the role of extracellular HSPs and their potential use as diagnostic and therapeutic tools in fibrosing interstitial lung disease (Figure 2 and Table 2). HSPs are ubiquitous proteins whose expression is enhanced under various stresses and diseases. HSPs have been demonstrated to be involved in numerous pathologies including fibrosing ILDs. Despite the growing body of evidence demonstrating that HSPs are largely involved in fibrosing ILDs, the diagnostic or therapeutic strategies involving HSPs’ measurement or inhibition/upregulation are far from being investigated. In addition, this review uncovers autoantibodies against eHSPs as potential biomarkers as they are found in many patients with ILDs. Nevertheless, little is known about their functions and whether their upregulation in fibrogenesis is a driving factor or if an indirect marker of the evolution of disease remains unclear. Antibodies against HSPs are found in normal conditions as well as in pathological contexts and they are described to modulate positively or negatively the immune response. Further investigations are required to understand whether they are a cause or a consequence of the disease and if they could be used as a diagnostic or therapeutic tool in fibrosing ILDs (Figure 2 and Table 2). It is clear that HSPs seem involved in all ILDs and results presented here clearly point out their role in pathogenesis as well as their presence in several biological fluids. Nevertheless, the major limitation in the use of eHSPs as biomarkers may be the lack of specificity of eHSPs since there are a wild range of conditions in which they are overexpressed. However, a growing number of studies supports their usefulness, and options for improvement exist by combining several markers. In addition, even though the use of eHSPs as biomarker of diagnosis in the context of ILDs seems unlikely, their use as biomarker of disease progression and/or therapy efficacy in fibrosing ILDs may be useful to improve patients’ outcome and management.

Moreover, strategies in vivo to modulate eHSPs’ expression were somehow effective to prevent, limit or stop the progression of certain types of ILDs such as IPF. Finally, the current review highlights the lack of investigations on the role of extracellular vesicles and especially HSPs in EVs in the field of fibrosing lung diseases. Today, extracellular HSPs and, in particular, HSPs in exosomes are widely studied for their theranostic potential in cancer. It is important for the respiratory research and clinical field to take advantage of this knowledge about HSPs in exosomes that are the reflection of eHSPs from cells and try to discover new therapeutic targets and diagnostic strategies to overcome the burden of lung diseases that continues to increase over the years.

## Figures and Tables

**Table 1:** HSP nomenclature and localization.

Classification	Nomenclature	Weight Discrimination	Localisation	ATP Depending
**HSP27**	HSPB1	Small molecular weight	Cytoplasm/nucleus	ATP-independant
**αB-Crystallin**	HSPB5		Cytoplasm/nucleus	ATP-independant
**HSP90**	HSPC	High molecular weight	Cytoplasm/Endoplasmic reticulum/Membrane	ATP- dependent
**HSP70**	HSPA1A		Cytoplasm/nucleus/mitochondrie/endoplasmic reticulum	ATP- dependent
**HSP47**	DNAJ		Endoplasmic reticulum	ATP-independant
**HSP60**	HSPD1		Mitochondrie	ATP- dependent
**HSP65 Mtb** **HSP16 Mtb** **HSP70 Mtb**	DNAK		Membrane/cytosol/envelope	ATP- dependent
**Alpha-2-HS glycoprotein**	Fetuin-A		Cytosol	ATP-independant

**Table 2:** Incorporation of pathogenic roles of eHSP in ILDs that implicate their roles as biomarkers and as therapeutic molecules.

Pathology	eHSP	Protein	Auto-Antibody	Biological Fluids	Prognosis/Diagnosis	Authors
**IPF**	HSP90	+	−	Serum	Prognosis	[16]
	HSP70	−	+	Serum, BALF	Prognosis/Diagnosis	[38,39,40]
	HSP47	+	+	Serum	Prognosis	[41,42,43]
**SSc**	HSP70	+	−	Serum, BALF	Prognostic	[44,45]
	HSP90	+	−	Blood	Prognostic	[46]
	HSP27	+	−	Blood	Diagnosis	[47]
	α2 HSP	+	−	BALF	Diagnosis	[48]
**RA**	HSP90 citrunillated	+	+	Blood	Prognosis/Diagnosis	[49]
	HSP70	+	−	Blood	Under investigation	[50]
**SA**	HSP70	−	+	Serum	Diagnosis	[51]
	HSPMtb 16	+	−	Serum	Diagnosis	[52]
	HSPMtb 65	+	−	Serum, BALF	Diagnosis	[53]
	HSPMtb 70	+	−	Serum	Diagnosis	[54]
	HSP70	+	−	BALF	Diagnosis	[53]
	HSP90	Deserve further investigated in fibrosing ILD context				
	HSP60					
**NSIP**	HSP47	+	+	Serum, BALF	Diagnosis/Prognosis	[55]
						[42]
						[56]
**PNC**	HSP70	+	−	Serum	Diagnosis	[57]
	HSP27	+	−	Serum	Diagnosis	[58]
	HSP90	+	−	Blood	Under investigation	[59]
	HSPMtb	Deserve further investigated in fibrosing ILD context				
**HP**	HSP70	+	−	BALF	Diagnosis	[60]
	α2 HSP	+	−	BALF	Diagnosis	[61]
	HSPMtb 65	Deserve further investigated in fibrosing ILD context				
**DILD**	HSP47	+	−	Serum	Diagnosis	[62]

## Data Availability

Not appilcable.
